# Developing dimensions for a multicomponent multidisciplinary approach to obesity management: a qualitative study

**DOI:** 10.1186/s12889-017-4834-2

**Published:** 2017-10-16

**Authors:** Anita J. Cochrane, Bob Dick, Neil A. King, Andrew P. Hills, David J. Kavanagh

**Affiliations:** 1 Institute of Health and Biomedical Innovation, School of Exercise and Nutrition Sciences, Faculty of Health, Queensland University of Technology, GPO Box 2434, Brisbane, QLD 4001 Australia; 2 Interchange, 37 Burbong Street, Chapel Hill, Brisbane, QLD 4069 Australia; 3 Institute of Health and Biomedical Innovation, Faculty of Health, Queensland University of Technology, GPO Box 2434, Brisbane, QLD 4001 Australia; 4 School of Health Sciences, Faculty of Health, University of Tasmania, Locked Bag 1322, Newnham Drive, Launceston, TAS 7250 Australia; 5 Institute of Health and Biomedical Innovation, School of Psychology and Counselling, Queensland University of Technology, GPO Box 2434, Brisbane, 4001 Australia; 6 LifePsyche, Box 3180, Norman Park, Brisbane, PO 4170 Australia

**Keywords:** Action research, Convergent interviewing, Multicomponent, Multidisciplinary, Obesity, Weight management

## Abstract

**Background:**

There have been consistent recommendations for multicomponent and multidisciplinary approaches for obesity management. However, there is no clear agreement on the components, disciplines or processes to be considered within such an approach.

In this study, we explored multicomponent and multidisciplinary approaches through an examination of knowledge, skills, beliefs, and recommendations of stakeholders involved in obesity management. These stakeholders included researchers, practitioners, educators, and patients.

**Methods:**

We used qualitative action research methods, including convergent interviewing and observation, to assist the process of inquiry.

**Results:**

The consensus was that a multicomponent and multidisciplinary approach should be based on four central meta-components (patient, practitioner, process, and environmental factors), and specific components of these factors were identified. Psychologists, dieticians, exercise physiologists and general practitioners were nominated as key practitioners to be included.

**Conclusions:**

A complex condition like obesity requires that multiple components be addressed, and that both patients and multiple disciplines are involved in developing solutions. Implementing cycles of continuous improvement to deal with complexity, instead of trying to control for it, offers an effective way to deal with complex, changing multisystem problems like obesity.

## Background

There is a global pandemic of obesity [1]. Despite the distribution of numerous national and international obesity management guidelines [2–5], and notwithstanding isolated examples of success in managing the condition e.g., [6], no nation has provided compelling evidence of reversing its obesity epidemic [7].

To solve the refractory nature of obesity, a recurrent recommendation is for a stronger evidence base for managing the condition [8–10]. It is true that current behavioral interventions have relatively small average effects on weight, and while bariatric surgery gives stronger average effects, it does so at the risk of negative sequelae and high cost [11]. However, the large and increasing body of publications on obesity [12] without a commensurate change in its prevalence suggests an increase in the extent of controlled trials is not providing an answer [13, 14].

A key issue in this lack of traction in addressing obesity may be the ability to translate the existing research into practice. A survey of dietetic practice in weight management provides evidence of the gap between research and practice in obesity management. The survey was conducted 6 years after the release of the 2005 Dietetic Association of Australia (DAA) Dietetic Best Practice Weight Management Guidelines for Overweight and Obesity in Adults. It found that fewer than half of the respondents had read the guidelines in full. Only one in ten had attended a professional development event that trained them in how to properly implement the guidelines [15].

A temptation may be to solely blame a lack of evidence translation on practitioners—if the problem may be solved by better training about the evidence and related practice guidelines, better supervision, or increased cues and incentives for them to implement evidence-based practice would be required. As important as these factors may be, another potential reason is the applicability of the research they are asked to implement. Whether a particular treatment achieves statistically significant outcomes in a research trial does not necessarily mean that these outcomes can be replicated in naturalistic settings [16, 17], not only because of the potential for lower fidelity of the treatment’s application in those settings, but also because of contextual differences (e.g., less available staffing and other resources) and differences between trial participants and the users of the practitioner’s service. Weight loss trials typically implement a standard intervention without substantial individual tailoring, and translating research evidence to the treatment of individual patients who differ markedly from participants in a specific research study presents a significant challenge [18]. In addition, most weight loss treatments were developed by researchers without consulting practitioners or patients [8, 19, 20], which runs the risk of them being sub-optimal in their acceptance and impact.

Recognition of the complexity of drivers associated with obesity is reflected in recommendations identified in position papers [10, 21] and reviews [22–25] for multicomponent (MC) and/or multidisciplinary (MD) interventions as the treatments of choice for obesity management. However, despite endorsement of MC and MD approaches for obesity management by obesity experts, reviews and position papers have not identified specific recommendations for operationalizing MC and MD approaches.

There is consensus that obesity is a complex and multisystem condition and therefore unlikely to be solved using simplistic causal models [26, 27]. This point is supported by the greater long-term impact from the combination of diet and exercise, and of behavioral and surgical methods, that form single components [11, 28, 29]. However, multiple components and multidisciplinary collaboration may still not be enough. Solely relying on highly controlled randomized controlled trials with individual patients to inform practice may not be optimal in addressing a phenomenon whose determination is highly complex, individually variable and subject to environmental and social change [30, 31]. A more effective approach may be to encourage practitioners to integrate evidence-based practices derived from experimental studies with the practice-based evidence they collect during routine practice [17, 32]. Treatments that are co-designed with end users, are individually tailored, and are modified dynamically according to patients’ responses, are likely to have greater impact than ones that do not have these features [33].

### Thematic concerns and aim

We aimed to make progress towards addressing the intractability of obesity, the gap between theory and practice among practitioners, limitations of using narrowly defined evidence-based treatments for a condition that is complex and multi-systemic, and the lack of clarity in how multicomponent, multidisciplinary (MCMD) approaches could be more effectively applied in practice.

The specific aims of this research were to operationalize a MCMD approach for obesity management and to develop a theoretical model that could guide the approach. The model and approach were intended to guide practitioners in generating sound practice-based evidence and integrating that evidence with research. We expected that this would address the gap between evidence-based recommendations and routine practice [13, 14], by encouraging practitioners to adopt a critical perspective both towards their own experience and their review of the evidence-based literature.

In consideration of the concern that approaches to obesity management are often formulated by people who do not engage with end users directly, we planned that our current treatment model would be informed by a variety of stakeholders, including researchers, practitioners, educators, and patients.

## Methods

### Methodology

We did not identify any literature that clearly outlined a theoretical framework for a multicomponent and multidisciplinary approach to obesity management, so regarded the current research as exploratory. Rao and Perry [34, 35] have identified action research techniques as amenable to pilot or exploratory research, because action research derives emergent theory from the data collected. By using action research methods, we would be able to capture the nonlinear complexity of obesity from a real-world perspective and design and evolve a MCMD approach that was responsive to information and experience generated by the research process over time. Consequently, we chose action research as a theoretical and methodological framework.

A benefit of using action research as a theoretical framework for this study was its ability to function as a meta-process under which other methods for data collection can be managed [36]. In utilising this function, this study drew data from interviews, observation and the literature. By triangulating the interview data with observational data and the literature, we were able to corroborate and elaborate on the thematic concern of developing a MCMD approach to obesity management.

### Procedures

#### Convergent interviewing

We used convergent interviewing as the main source of data, because it is both an interview technique and a process for data interpretation [37]. The technique consists of a series of long, in-depth interviews that gather unstructured content, while using a structured process for the interviews and for data analysis [35]. The cycles of data collection and analysis facilitate the development of shared epistemological and ontological understandings among the disciplines involved, thereby overcoming challenges inherent in projects involving multiple disciplinary perspectives [38].

The criterion for qualitative sampling is not the sample size but rather case contrast [39], and also data saturation [40]. To achieve a representative sample with maximum diversity to interview, we conducted a stakeholder analysis process [40]. The process involved the research team generating a list of stakeholders who would be interested in, or affected by, the thematic concerns. From this list we chose interviewees who had the relevant subject knowledge for our research and who were as different from each other as possible [41]. We chose the stakeholders from a broad subject pool to ensure adequate consultation regarding the factors to include in a MCMD approach to obesity management. Interview data consisted of their reflections and interpretations of their own practice in obesity management or their personal weight loss experiences.

When using convergent interviewing, Dick [42] suggested a minimum of 12 interviews to ensure saturation in the perspectives that are provided as the data converge. Others have found that saturation can occur earlier [43]. We chose 14 stakeholders to be interviewed. They included, in the sequence they were interviewed: a dietician nutritionist (DN), an obese patient with a health background (P1), a general practitioner (DR1), a counselling psychologist (PP), a morbidly obese patient (P2), a social worker (SW), a research psychologist (PR), a naturopath (N), an endocrinologist (DR2), a health epidemiologist and behavioral biologist (HEp), a team leader of a community-based health promotion team (N2), two community health nurses (N1), an exercise physiologist (EP), and a medical educationalist (MEd).

The first author conducted the interviews. The person deemed to be the most representative of the target population, DN, a dietician nutritionist, was interviewed first. DN had worked in clinical practice, research, education, government policy, and as a national representative of her profession. The breadth of DN’s knowledge and opinion formed a broad platform for subsequent interviews. A patient, P1, was interviewed next. She was representative of the target population, but in other respects unlike DN. P1 had struggled with her weight for more than 20 years and had been unsuccessful in losing weight despite using numerous approaches to weight loss. The general practitioner, DR1, was the third interviewee because she was the next most representative member of the target population, but was unlike the first two, and so the sequence continued.

Convergent interviewing was chosen because the interviewer is not required to make a priori assumptions about which questions to ask. The process began with a broad initial question: “What do you believe needs to be included in a weight management assessment or approach to optimize outcomes or ensure success?” Responses of those interviewed were used to generate the themes for questions to be posed in later interviews, so we could establish confirmatory or disconfirmatory evidence from the data collected. As Dick [40] proposes, convergence occurs not only over the series of the interviews, but within each interview itself. Each interview terminated when no more information was generated; a pattern was evident in the data; convergence with previous interviews had been confirmed or disconfirmed; and discrepancies were explained, where possible.

An iterative analysis of data commenced at the same time as the first data were collected. We used a computerized qualitative data management system, nVivo (QSR, N8, 2010), to construct conceptual frameworks from the large volume of data generated by the convergent interviews. This thematic analysis was achieved by analyzing the data and breaking them down into interpretable and meaningful categories, often referred to as conceptual “codes” or “nodes” [44]. Researcher bias is reduced when convergent interviewing processes are employed, because participatory data analysis is inbuilt. A component of convergent interviewing is to invite participants to explain disagreements with earlier interviews so that they are helping to interpret the data collected. To further reduce researcher bias, members of the research team reviewed the data analysis. The data analysis was also presented and discussed with groups of health professionals drawn from multiple disciplines, which the primary author facilitated. At all times, to mitigate or minimize the risks of bias, we paid attention to any evidence from any source that appeared to challenge or disconfirm our ideas.

##### Free nodes

Iterative analyses of data from the interviewees allowed us to identify patterns and generate explanations for phenomena. We commenced by sorting the first interviewee’s data into free nodes. A property of a free node is that it represents a theme evident in the data but does not presume relationships or connections with other nodes.

##### Tree nodes

As we progressed with individual analysis, free nodes that clustered together became more obvious and we sorted and grouped them into categories called tree nodes [45]. In each successive action research cycle, codes were reassigned, collapsed, renamed and deleted to further clarify the data and to create meaningful, understandable, and trustworthy categories that explicated a MCMD approach to obesity management. As the interviews cycled, we converged from a tentative interpretation of the data in early interviews to a clearer and more stable interpretation of the constructs and processes relevant to a MCMD approach by the time of the final interview [34].

#### Observational procedures

Observation in the “real world” helped to provide insight into the thematic concerns and build a picture of the context in which the research problem existed [44]. Observation procedures involved recording (written and audio), analyzing, and interpreting people’s actions and interactions [37] in a wide variety of settings.

We conducted most of the observational data collection in group settings, with a series of observational events being explicitly dedicated to having stakeholders put forward their ideas for developing a MCMD approach to obesity management. Settings used as part of this research included multidisciplinary network meetings that the primary researcher either facilitated or participated in; talks she gave on obesity, including questions and comments from the participants; multidisciplinary team meetings and obesity talks she attended as an observer; obesity training events and conferences; and conversations she had with colleagues, including her doctoral supervisors, and patients. Field notes were either taken during the observation or written up immediately after the event to improve accuracy [46]. On several occasions, permission was requested to audiotape the observational session; these tapes were later transcribed. To promote consistency, we only used observational data that were representative of most patients.

#### Literature review

A review of the obesity literature, was conducted and this formed the basis for the thematic concerns on which the research was based. We used the EBSCO database and explored terms relating to the effectiveness of variants of individual weight management interventions, health promotion strategies, health care models and recent thinking about participatory and multilevel approaches to obesity management. In addition, consistent with the practice of action research [47], reference to the literature progressed as an ongoing dialectic, between the data and the literature, throughout the study. We used this dialectic to clarify, augment, challenge, and inform the data and the direction of the approach in development. Our review of the literature, after data collection and analysis, also identified how the research was adding to and contributing to current knowledge in the area under study [48].

#### Triangulation of data

The process of action research involved the collection of data through interviews (action) and the subsequent analysis of that data in light of theoretical (literature review) and practical considerations (observation). Distilling the data through successive action research cycles facilitated sense-making, model building and theory development. The use of multiple methods facilitated triangulation of the data and optimised rigor. By triangulating the interview data with observational data and the literature, we were able to corroborate and elaborate on the aim of developing a MCMD approach to obesity management.

## Results and discussion

A preliminary step in the research analysis was to determine whether or not stakeholders believed that current obesity approaches have not been effective. A second step was to identify whether stakeholders endorsed the development of a multicomponent multidisciplinary (MCMD) approach for obesity management as recommended by position papers e.g., [25].

### Justification for the research

Stakeholders endorsed the importance of clarifying a MCMD approach to obesity management as a focus for the current research. Eleven of the 14 interviewees reported disillusionment with current weight management strategies. The remaining three interviewees did not provide an opinion. The general practitioner, DR1, said, “I don’t think that our current interventions are actually doing anything.” The endocrinologist, DR2, agreed: “We’ve all got buckets of patients who’ve tried dieting and it hasn’t worked. Obesity clinics around the globe will tell you the same story.” The dietician, DN, further concurred: “Public health messages don’t work. Nothing has worked. We have got worse.” The second patient, P2, offered a patient perspective: “I’ve tried everything. If anything was going to work, it would have.”

When scanning the observational data, we were unable to find any disconfirming evidence for the consensus generated by the convergent interviews regarding disillusionment with current obesity management approaches. Similarly, we identified scant support for the effectiveness of individual-focused weight management approaches in the literature reviews [9, 49–51]. Although small weight reductions were noted in some reviews e.g., [52], weight regain was common [53]. In many of the reviews, the heterogeneity of study designs was concluded to have been a factor hindering the generalization of outcomes [52, 54].

The majority of stakeholders and observational data supported position papers and obesity guideline recommendations for pursuing MC and MD approaches for weight management. Two interviewees did not support a MCMD approach. The first patient we interviewed, P1, said she preferred to consult only a dietician for weight management. DR2 was the only practitioner who did not support a MCMD approach. DR2 said, “I think a MD approach is overcomplicating a simple issue.” DR2 believed all general practitioners (GPs) conducted medical histories of their patients, according to NHMRC guidelines, as a regular practice. However, both patients, (P1 and P2), countered DR2’s claim when they complained about short consultation times that hampered GPs being able to address their weight issues. “You are in and you are out,” said P2.

### Making sense of the data

As mentioned previously, data from the first interviewee, DN, were coded as free nodes. For example, an analysis of the first interview conducted with DN, revealed a likely free node to be ‘practitioner barriers’ to obesity management. Sample comments made by DN that led to the creation of the ‘practitioner barrier’ category included: “The doctor won't use anything that is not fast”, “Dieticians are lucky to get one week of training in obesity”, and “Most allied health professionals are overweight themselves.”

The contents of the free node, ‘practitioner barriers’, were related to practitioner factors, so it was decided to house this free node within an overarching category we termed a metacomponent. We labelled this metacomponent or tree node *practitioner factors*. We identified other comments made by DN as more process-oriented and created another metacomponent called *process factors*. This established a second tree node. We labelled one free node under this tree node ‘practitioner processes’. We evolved this free node from factors DN viewed as either unhelpful or helpful approaches for obesity management. Sample statements by DN that were reflective of ‘practitioner processes’ included: “Diet histories were great when everyone ate a standard type of meal”, and “Get into a partnership system with the patient where the patient has ownership, a self-management approach.”

DN’s focus during her interview reflected her more recent work history as a tertiary educator, researcher, and policy influencer. Her clinical experience with patients occurred early in her career, which might explain why she did not focus on patient-related issues during her interview. We determined that the content of ensuing interviews either converged with or diverged from DN’s viewpoint, contributing to the evolution of the tree nodes and the development of a MCMD approach. Summary themes were refined iteratively as more data were entered.

The second interviewee was patient 1, P1. We created a free node called ‘patient barriers’ to weight loss based on P1’s comments. Sample comments justifying the inclusion of patient barriers included: “When things are going bad, I hit the fridge or I comfort eat”, “I eat unconsciously”, and “It’s a vicious cycle.” Based on P1’s comments we introduced an additional metacomponent, *patient factors*. P1’s comments both converged with and diverged from comments made by DN, substantiating free nodes and their placement under metacomponents, and introducing new understandings and concepts. For example, unlike DN, P1 viewed dieticians as the obesity experts, but agreed with DN that doctors were not experts in weight management: “I don’t think the doctor is the expert. I think they are too busy. I think a dietician is the expert in the field.” P1’s comments endorsed DN’s view that practitioners needed to ask patients what they want. This led to the creation of a free node, ‘person-practitioner fit’, which we placed under the metacomponent, *process factors*. P1 clarified what she expected from a practitioner: “I want someone to really listen and not be judgmental”, “You need encouragement, understanding, empathy, kindness”, and “To be treated as an individual.” P1’s comments supported the free node, ‘practitioner processes’, which we had already conceptualized from DN’s comments, and assigned to the metacomponent, *process factors*.

Ensuing interviews converged with or diverged from the preceding interviewee’s viewpoint, contributing to the evolution of a MCMD approach. We achieved data saturation for the four metacomponents within the 14 interviews that were conducted. It is intended to elaborate the categories within these metacomponents during the actionable phase of the model. However, we believe that we have gained enough information for an initial structure on which to develop a MCMD approach.

### Developing the MCMD approach for obesity management

Below is an analysis and interpretation of the tree nodes we used to evolve the framework for the MCMD approach using NVivo.

#### Metacomponents

In the early stages of the action research process, three central themes were evident in the interview data and were labelled: *patient factors*, *practitioner factors*, and *process factors*. We referred to these overarching factors as metacomponents. These metacomponents mirrored the data sources (patients and practitioners) and their ideas for approaches to weight management (processes). A subsequent reference to the literature showed that these same components were also distilled in a Cochrane Collaboration review investigating MC interventions for diabetes [55].

#### Components

To improve the conceptual clarity of the nodal hierarchies, the tree nodes positioned under each metacomponent were referred to as *parent components* [45]. Parent components housed another layer of subcategories referred to as *child components*. This process continued with child components spawning a layer of grandchild components, and so on. The progressive and deeper levels of generational analysis illustrated the multifaceted complexity of obesity. However, as a way of managing the large volume of data, only a summary of the parent components for each metacomponent have been included in this article.

When stakeholders were invited to specifically identify what they believed were the most important components to include in a MCMD approach to obesity management, they nominated the following parent components, in order of emphasis: ‘psychology’, ‘food and nutrition’, ‘physical activity’, and ‘medicine’. These components substantiated the inclusion of *patient factors* as a metacomponent and reflected the central role attributed to the patient in current weight management initiatives. This outcome also provided insight into the mental model stakeholders hold in relation to multicomponent approaches to obesity management.

The interviewees did not mention the other three metacomponents (processes, the practitioner or the environment), or their subcomponents, as specific components for a MCMD approach. The overall pattern of the metacomponents and parent components we present below were distilled from a comparison of the interview data and observational data and the relevant literature. Because we used an action research methodology, we assumed that the components nested under each metacomponent would be modified through successive cycles of inquiry and action within both the current research and in future work. The deconstruction of data taking place throughout this study forms a starting point only.

#### Parent components for the patient factors metacomponent

The *patient factor* metacomponent was composed of the components directly relating to the patient. They included ‘psychology and social’, ‘diet and nutrition’, ‘bio-medical’, ‘health behaviours – eating behaviour and physical activity’, ‘demographics’ and ‘weight-related’ factors. Some other components were considered relevant (e.g., finances) but were not as prominent in the data and are therefore not explicated further in this paper.

The purpose of briefly surveying the following parent components is to justify and explain the component’s inclusion and to begin building a picture of what a preliminary MCMD model for weight management could be.

##### Psychology and social components

Most stakeholders firmly supported the inclusion of ‘psychology’ as a component in a MCMD approach. The research psychologist, PR, said, “Psychology has a massive place in obesity research and treatment. It is one of the major causes of obesity.” The psychologist in private practice, PP, believed “everyone with a BMI over 30 should be referred to a psychologist”. A patient we interviewed echoed the sentiments of the psychologists: “I think the psychological is something that is really missing; my weight gain is definitely psychological” (P1). The majority of stakeholders who attended observational sessions also strongly believed ‘psychology’ and psychologists should play a role in obesity management.

The two stakeholders we interviewed that did not endorse ‘psychology’ as a component were the medical practitioners. Neither DR1 nor DR2 referred their obese patients to psychologists. DR1 had not considered it. However, DR2 was not supportive of psychological interventions because of his belief that “patients fundamentally don’t have any interest in losing weight.”

When we reviewed the literature, we identified a number of areas that supported the inclusion of ‘psychology’ in obesity management. These include: eating and stress [56], self-control and food consumption [57], binge eating and associated cognitions [58], depression and obesity, and their relationship to physical health problems [59], weight perception and distress [60], relapse prevention and problem solving for weight maintenance [61], and personality factors in obesity [62].

‘Social’ factors also have a powerful influence on our food choices and eating habits. This influence justified their inclusion in a MCMD model. For example, interruptions to self-care caused by events such as injury or illness, financial and living insecurity, as well as institutional care, are factors that affect one’s ability to manage one’s food intake [63]. Social support has been identified as an important factor in facilitating weight loss outcomes [64, 65]. The status of family support needs to be addressed during assessment, particularly in the case of children and adolescents [66].

Observational data also supported the importance of ‘social’ factors in obesity management. Clients viewed eating as an important component of their social interactions and a major barrier to weight loss.

##### Diet and nutrition

There was agreement between all stakeholders and across the reviewed literature e.g., [2, 10, 23, 67, 68] that ‘diet and nutrition’ would be a component of a MCMD weight loss approach. We were unable to identify any information that did not support including ‘diet and nutrition’ as a component in a MCMD approach.

##### Bio-medical

The practitioners with medical backgrounds (i.e., nurses and doctors) openly supported the inclusion of ‘bio-medical’ factors and doctors in a weight management approach. However, the interviewed patients and members of non-medical disciplines did not underscore the role of ‘bio-medical’ factors in weight management. Their focus was on behavior, diet and exercise.

Theoretically, a medical examination, particularly in cases of severe obesity, should assist the practitioner to identify any physiological or pharmacological causes of obesity, and assess health risks including the presence of weight-related comorbidities such as diabetes mellitus type 2, sleep apnea, insulin resistance and hypertension [69, 70]. Medical assessments are also required for assessing cardiorespiratory fitness and screening for musculoskeletal issues (e.g. arthritis) prior to physical activity prescription [25]. The place of ‘bio-medical’ factors in weight management is therefore well justified.

##### Health behaviors

The majority of stakeholders acknowledged that ‘physical activity’ was a necessary component of a weight loss program. Our review of the literature also supported the inclusion of ‘physical activity’ as a component in a MCMD approach [71–74]. However, exercise was the least understood of the components of obesity by the interviewees. The exercise physiologist (EP) stated, “Very few people understand the energy balance model and understand the energy of exercise.” Stakeholders were also not in agreement as to whose role it was to deliver exercise advice. A patient said, “I wouldn’t want to go to an exercise physiologist … just by the name, he’s just into exercise ... he really wouldn’t be interested in weight loss.” (P1). A doctor added, “It seems to me what the exercise physiologist takes on as his capacity would be what I take on as my capacity. (DR1)”.

Interview and observational data also highlighted the relevance of including ‘eating behaviors’ in the MCMD model. Stakeholders identified their issues with food and eating behaviors as reasons they gained weight and could not maintain weight loss. Poor ‘eating behaviors’ cited by stakeholders included over-eating, bingeing and emotional eating.

Triangulation with the literature supported the inclusion of ‘eating behavior’ as a component for a MCMD model. The World Health Organization [75] has recognized poor ‘eating behaviors’, combined with sedentary lifestyles, as drivers of obesity. As pointed out in reports such as those provided by the National Health and Medical Research Council [76] and Foresight Report on obesity [77] there is a complex interplay between energy intake and energy expenditure behaviors that are worthy of exploration in managing obesity. The evolution of an obesogenic environment has made over-eating easy [78] and contributed to the increase in disordered ‘eating behaviors’ [79].

##### Demographics

‘Demographic’ factors were not strongly identified as a stand-alone component for a MCMD approach by the stakeholders we interviewed. However, ‘demographic’ factors were distilled as a parent component during the nVivo analysis and triangulation with observation and the literature. Subcomponents of the ‘demographic’ parent component, which we referred to as child components, were also clearly identified as factors that could inform obesity management. These included, gender [80], occupation and education [81], ethnicity [70], and age [82].

##### Weight-related

‘Weight-related’ factors are a face valid inclusion in any model for obesity management. Preliminary components distilled for this component included: barriers to weight loss, monitoring, follow-up, anthropometry, achievement, weight goals and weight history.

#### Parent components for practitioner factors metacomponent

The parent components for the *practitioner factors* metacomponent included: ‘roles and boundaries’, ‘resources and barriers’, and ‘patient-practitioner fit’.

##### Roles and boundaries

The parent component, ‘roles and boundaries’, arose from several stakeholders’ comments. The medical educationalist, MEd, explained that he scoured the literature to find the best evidence for producing change. His search led him to the conclusion that “changing clinician behavior related to better patient health outcomes.” This finding is supported by work in psychotherapy [83]. The dietician, DN, believed ‘roles and boundaries’, if not addressed, would be the most significant practitioner barrier in successfully implementing a MCMD model for weight loss. The nurses we interviewed, N1 and N2, concurred.

##### Resources and barriers

The ‘resources and barriers’ parent component contained items referring to practitioner abilities and constraints, which could affect a practitioner’s ability to treat obesity and achieve positive outcomes. Numerous barriers to a MCMD approach were reported. Notably, acknowledgement of a “silo mentality” by stakeholders in the current research was captured in a number of statements. MEd summarized, “Clinicians have a silo mentality”. The psychologist in private practice agreed: “I don’t know how to work outside my own silo” (PP). A nurse who worked in a multidisciplinary team commented: “You see some people who just sit in their discipline and feel like they shouldn’t be on a multi-disciplinary team. It’s the person who’s really about the multi-disciplinary team and sees the value in the multidisciplinary team rather than being in a discipline specific team that you need” (N1).

A silo mentality is a current challenge in healthcare, because it makes it difficult to achieve integration and collaboration among the disciplines providing services to the patient [84]. McNair [85] summarized, “A silo approach to education; distinct professional codes of ethics; and the drawing of boundaries around uni-professional knowledge, all undermine respectful awareness of knowledge and skills of other disciplines and fuel interdisciplinary rivalry” (p. 3).

Kreindler et al. [84] argued that “The success of health reform stands on the ability of delivery system reform to replace fragmentation and waste with coordination and cost-effectiveness.” (p. 348). To achieve this, Bammer [86] argued that strategies to achieve an integration and collaboration across “silos” must target the micro level of inter-professional teams and the macro level of healthcare organizations.

##### Patient-practitioner fit

‘Patient-practitioner fit’ refers to the quality of the relationship between the patient and his or her practitioner. A closely related concept is referred to as the therapeutic or working alliance [87]. Of the three parent components for the *practitioner factor* metacomponent, ‘patient-practitioner fit’ received the strongest support in the convergent interviews. The two patients who were interviewed endorsed the importance of therapeutic alliance. They stressed that “liking” their practitioner was a prerequisite for retention in weight loss programs. Published studies also confirmed that personality congruence between the patient and the practitioner optimizes alliance [88]. Inclusion of the ‘patient-practitioner fit’ parent node was further supported by numerous studies that indicate that the therapeutic or working alliance is one of the strongest predictors of therapeutic outcomes [88]. Optimizing patient-practitioner fit also reduces psychotherapy treatment dropout [89].

Although there is a significant body of research papers on working alliance and psychotherapy outcomes, there are very few published articles on working alliance and obesity. An exception was a study that explored patient-practitioner relationships and obesity management outcomes [90]. This study found that weight loss outcomes were adversely affected when the patient-practitioner relationship was disrupted.

#### Parent components for process factors metacomponent

The *process factors* metacomponent contained processes that assist in tailoring the MCMD approach and implementing the interventions in a dynamic manner that is responsive to the patient’s condition and situation at any given time. Implementation of these processes would promote ongoing engagement by the patient and optimize coordination of treatment across the disciplines. Process factors were divided into the three parent components: ‘patient processes’, ‘practitioner processes’, and ‘team processes’.

##### Patient processes

Patient processes involved the steps employed to assist the patient to self-manage. DN said:

Public health approaches fail because they do not engage at all with the public. Self-management approaches engage with the patient. You have a therapeutic relationship, but the patient has ownership. Overweight is so complex. If you don’t have the patient generating the suggestions and a counsellor asking ‘Why do you do this? How do you get your food?’, you won’t engage the patient and give them ownership.

A nurse agreed, “We promote self-management and patient focus and ask questions like, ‘what is your problem and how can we work together to get over it?’” (N1). The medical educationalist suggested: “Train the clinicians in the skills they need to help the patients self-manage. Promote partnership between clinician and patient” (MEd). Similar to the dietician and nurse, MEd believed this approach put both the practitioner and the patient “on the same page” and thereby optimized outcomes.

Our health care systems were historically predicated on acute conditions in which the patient played a passive role, and were not designed to meet the needs of chronic conditions like obesity, which require multidisciplinary treatment and self-management approaches [91, 92]. People with chronic conditions grapple with the physical, psychological, and social pressures imposed by their conditions, and according to Wagner et al. [91], need integrated care models to help them to self-manage their illnesses. The practice of reducing patients to a diagnostic classification and applying a pre-formulated weight-loss practice has not translated into positive weight management outcomes [49, 50]. As outlined in various self-management guidelines [93, 94], patients are likely to benefit from having the information, skills, confidence and motivation to interact with their health care team and work with their treatment program. As Lambert, Garfield and Bergin [95] emphasize, “Clients are not inert objects or diagnostic categories on whom techniques are administered. They are not dependent variables on which independent variables operate. People are agentive beings who are effective forces in the complex of causal events” (p. 814).

##### Practitioner processes

This parent node referred to the processes that practitioners preferred to use for weight management interventions. Most stakeholders supported a multidisciplinary approach. However, there was no clear agreement on who should perform the assessment and coordination of a MCMD approach. Both of the interviewed doctors believed it was the doctor’s role. However, the four allied health professionals held that the primary health care professional should perform the assessment, regardless of their discipline.

This diversity of opinion regarding MCMD approaches and obesity management in general was also reflected in observational data and the literature. Both the data collected during the interviews and the observations indicated that most practitioners do not use systems that collect, summarize and evaluate individual or cumulative patient data to inform care. The consensus was that dynamic approaches to obesity management that are responsive to both the practitioner and the patient could be effective. To manage chronic conditions effectively requires delivery designs that facilitate productive practitioner-patient interactions [96].

##### Team processes

‘Team processes’ are integral to team effectiveness [97]. The practitioners who worked in teams were the only stakeholders to expand on ‘team processes’ during the interviews. They included the nurses, social worker, the dietician and the medical educationalist who trained health professionals in health care processes. Practitioners who worked independently did not refer to ‘team processes’.

Due to insufficient collaboration between the participating practitioners, observational sessions did not result in the desired outcome of developing a MCMD approach. A “silo mentality” appeared to prevail.

The literature we reviewed on multicomponent and/or multidisciplinary studies for obesity management did not refer to ‘team processes’ when exploring the effectiveness of these approaches e.g., [98, 99]. However, there was a repository of information in the healthcare literature that provided conceptual frameworks to influence policy and practice that included a focus on teamwork. Examples included: “Creating a culture for interdisciplinary professional practice” [100]; “Promoting effective teamwork in healthcare” [96]; “A new health system for the twenty-first century” [92]; and “Transforming the delivery of health and social care” [101].

#### Parent components for environmental factors metacomponent

The advantage of triangulating the interview data with observational data and reference to the literature was that it facilitated deliberation on components that were considered important, but were not strongly emphasized by stakeholders during the interviews. The most outstanding example of this was the impact of *environmental factors* on the development and maintenance of obesity. Although we initially designated environmental issues as a parent component under the *patient factors* metacomponent, we also acknowledged that interviewees did not consistently refer to the patient’s environment as a consideration in weight management. The psychologist (PP) and social worker (SW), both of whom treated individual patients, referred to the patient’s social environment. Only the dietician, DN, the health epidemiologist (HEp), and the endocrinologist (DR2) referred to the broader environment. HEp encapsulated the relationship between obesity and the environment as follows: “Obesity is a signal that something is going wrong in the whole environment.”

Considering the prominent role that environmental issues play in obesity [77, 102], it was notable that most stakeholders did not focus on it. Neglecting to emphasize *environmental factors* could be attributed to the “creeping” nature of obesity, as suggested by HEp, and to practitioners providing data from the framework in which they worked, as suggested by DN.

Literature sources that explored more innovative approaches to managing obesity and chronic disease consistently incorporated an environmental focus [77, 91, 96, 102, 103]. The triangulation of stakeholder data with the literature suggested that *environmental factors* were significant enough to stand alone and be considered as a fourth metacomponent. This reinforced the advantage of using a methodology that took into account the perspective of patients, practitioners, the patient-practitioner interface, the literature, and other relevant data sources.

#### Final model

**Fig. 1:**
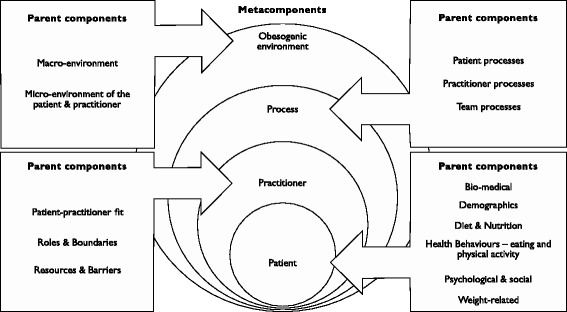
A multicomponent model for managing obesity

Feedback that was produced by iterative action research cycles provided both converging information and additional perspectives across multiple metacomponents and parent components. This allowed the derivation of a model of practice-based evidence that was also informed by evidence-based practice. Figure 1 provides a schematic representation of a multicomponent approach to obesity management based on the sum of the data collected. We nested the patient and practitioner metacomponents in the overarching metacomponent that we referred to as the obesogenic environment. Process factors facilitate interaction between these metacomponents and their parent components.

### Which disciplines should be included in a MCMD approach?

The scope of the parent nodes housed under the four metacomponents justified why multiple disciplines are necessary in effectively treating obesity. We analyzed the interview and observational data to identify which disciplines received the strongest support for inclusion in a MCMD approach. The roles receiving the most emphasis included the dietician, psychologist, exercise physiologist, and to a lesser extent, the general medical practitioner (GP). These roles mirrored the components identified as being the most relevant and important to include in a MCMD approach. Physiotherapists and nurses were given secondary emphasis in our data. There was only cursory reference to occupational therapists, complementary medicine practitioners and bariatric surgeons. During implementation, we would expect the team providing the treatment to recruit other practitioners as their inclusion became relevant.

We noted that practitioners appeared to interpret obesity through the lens of their own discipline. There was a lack of knowledge about other practitioners’ roles and how these roles could be applied in obesity management. The importance of including a range of disciplines in obesity management is evident in recent NHMRC obesity guidelines [10]. These guidelines list the types of practitioners and disciplines that could be involved in MD teams for obesity management as dieticians, exercise physiologists, specialist medical physicians, and general practitioners, as well as bariatric surgeons, psychologists, diabetes educators, social workers, occupational therapists, physiotherapists, aboriginal and multicultural health workers, general nurses, practice nurses, and mental health nurses. To optimize a MCMD approach to obesity management, barriers such as insufficient clarity about the potential roles of other practitioners, combined with a “silo mentality”, need to be considered [101].

### Developing a theoretical framework for a MCMD approach

The MCMD model we have developed endorses obesity as a complex problem that involves multiple actors and factors [104]. These multiple actors and factors interconnect either directly or indirectly making it impossible to depend on, and plan and prepare for the effect of any single intervention used in managing obesity for an individual [105]. Complex problems like obesity require multiple sites of intervention, unlike simple problems where the outcomes of actions are usually linear and predictable [106].

A limitation of current approaches to obesity has been the inability to incorporate a theoretical framework that integrates the multiple factors that impact on a person and their weight [26, 107]. When developing a theoretical approach to a problem, Tsui [108] emphasises the importance of accurately matching the theory’s premises to the issue being studied. Tsui argues that this increases the relevance of the research to practice as well as knowledge. Accordingly, one of our intentions was to develop a theory for the MCMD model we co-produced during this research. Developing a theory for the MCMD model could guide practitioners and patients in responding more dynamically and responsively to the complex and multi-systemic nature of obesity, and provide a framework for practitioners to conduct their own research on the application of the MCMD model in their practice.

Glaser [109] makes a distinction between emergent approaches for theory development, where theory arises from data, and forced approaches, where the research commences with a theory and forces the data to fit the theory. Given that “one size fits all” approaches do not work for complex conditions like obesity, we used a data-driven methodology, action research, on which to base the MCMD model. Fox [32] argues that theory building should be a necessary part of developing understanding but should be viewed more as an addition to practice not an end in itself. The advantage of using action research methodologies for a complex problem like obesity is that because it is an emergent methodology, it amasses understanding (actionable knowledge) gradually through iterative cycles of planning, action and reflection [110]. Dick emphasises that not only is the content (developing theory) emergent, but so are the processes or strategies. Dick also explains that data analysis, interpretation of data and theory building occur at the time of data collection. This feature allows the practitioner to derive theory from practice, responsively and in collaboration with the patient, in clinical settings [30]. The theory-practice axis can then be further elaborated by promoting a collaboration between evidence-based practice and practice-based evidence [32]. Furthermore, the participative qualities of action research facilitate shared understanding and commitment among stakeholders, which in turn motivates collaborative action [110].

The responsiveness of action research methods to the emerging needs of a person and situation is a valuable asset in an ever-changing world [111]. By teaching the practitioner and patient to be reflexive, they learn to access their own tacit knowledge to produce theories in action that are relevant to the prevailing context [112]. The intention is to optimise the “relevance” and “currency” of the model by teaching practitioners (and potentially and desirably their patients) how to put theory into practice and practice into theory. For a genuinely complex problem like obesity, it is difficult to know when the best solution has been reached. A key advantage of action research is that it allows the MCMD model to be refined through cycles of trial and error in establishing what works with different patients in various contexts.

In partial summary, it is evident that there are many different influences affecting the behaviour of a person seeking to reduce obesity. The literature relevant to these influences therefore spans multiple disciplines. Each patient is affected by her or his own life conditions and local environment. In addition, even an ideal treatment (if there is one) will work in many instances only with substantial commitment to it by the patient. Theory derived from the literature will contribute. However, by itself it is unlikely to be sufficient. There are more factors affecting obesity than any team of practitioners will understand fully. Further, there may be local conditions that the theoretical literature doesn’t address. Local practice-based evidence can be derived from the practice of each individual case. For good outcomes, it can be integrated with theoretical understanding from the literature. The following case study illustrates the unavoidable complexity of obesity treatment.

#### Case example

Judith presented for treatment of depression and morbid obesity with a psychologist and dietician. She was 65 years old and divorced. Her mobility was poor and she ambulated with a walking stick. She lived alone in a house her daughter owned. Judith reported feeling isolated, unwanted, useless and worthless. She had numerous medical issues, was anxious about her finances and was disillusioned with her efforts to find employment and lose weight. The MCMD approach was presented and collaborative goals established. Her first goal was to find more suitable housing that would also enable her sister to live with her. Strategies were also identified that would assist Judith in renting two pieces of real estate she owned in the country. Several real estate agents were engaged, and family members invited to assist her with this task. Completing these tasks reduced Judith’s financial pressure considerably and introduced social support. Judith then worked with her GP in relation to her medical issues, and consulted a number of specialists. Concurrent to working with her GP and psychologist, Judith self-initiated an energy controlled meal service and lost 15kgs. Her weight loss enabled her to walk without her walking cane. She reported accepting that she was retired and stopped pressuring herself to find work. However, over the Christmas period she relapsed and her depression rekindled. Using action research methodologies and the support of her practitioners, Judith was able to learn from her lapse and replan a path that would minimise the likelihood of relapse. She lost another lost 21kgs.

#### Reflection

The iterative cycles are a fundamental component of action research. “The cycle is a natural and logical way of responding to a complex and therefore uncertain situation that requires action” (B. Dick, personal communication, December 31, 2014); it parallels the way people problem solve a situation. In Judith’s case, she relapsed (an action), discussed her action with her practitioners then established a new plan to prevent relapse in the future (planning). This iterative facility within action research gave her permission to learn by doing and became a template for continuous improvement. Accepting that learning to lose weight through trial and error mitigates against the “all or nothing” thinking that is cited as a common reason for relapse [113]. Judith found the process self-empowering.

## Conclusions

Obesity is a chronic relapsing condition that has been resistant to resolution by non-surgical treatment approaches [114]. The intention of this research was to source problems and solutions reported by researchers, educators, policy influencers, practitioners and patients, to coproduce a MCMD approach to obesity management that addressed a number of practice issues or thematic concerns. Using qualitative action research methods, including convergent interviewing and observation, to assist the process of inquiry, we collected data that indicated multiple factors affect the energy imbalance underpinning the development of obesity. We housed these multiple factors under four metacomponents: practitioner factors, patient factors, process factors and environmental factors.

Psychologists, dieticians, exercise physiologists and general practitioners were most likely to be nominated as the practitioners to be included initially in a MCMD approach. However, there was a lack of general knowledge about one another’s role and how the roles could be applied in obesity management. The collective data emphasized that increased practitioner accountability in weight loss initiatives would likely benefit weight management outcomes. Both patients, P1 and P2, described how the interaction between the patient and practitioner influenced their decisions to either continue or discontinue weight management initiatives. During this research, comments were frequently made in relation to practitioner limitations, both by patients and practitioners during data collection. As recommended by the NHMRC guidelines [69], it is strongly recommended that any professional working with obesity undergo training or supervision with professionals who specialize in the area.

Based on the model we deconstructed from the data, we conclude that complex solutions will likely offer the best approach for a complex condition like obesity. Assessment procedures for a MCMD approach should have the potential to assess a broad array of causative and maintenance factors for obesity, which would provide the basis for interventions to be tailored to the individual [70, 115–117]. Furthermore, we believe the changing needs and circumstances of the individual over time suggest that a MCMD approach should have the capacity to endure uncertainty and unpredictability and be adjusted in an ongoing manner [20].

This research study demonstrated that dynamic and responsive methodologies like action research appear to lend themselves to the management of a complex condition like obesity. Implementing cycles of continuous improvement to deal with complexity, instead of trying to control for it, offers an effective way to deal with complex, changing multisystem problems like obesity [33]. As relapsing conditions [114], overweight and obesity require ongoing monitoring [70], and a close multidisciplinary collaboration would significantly help in ensuring that regular monitoring and early action on any lapse are in place.

The research forms the data collection phase of an action research cycle to develop and test a viable MCMD approach. We vigorously used processes that would identify any disconfirming evidence and thereby substantiate the efficacy of the research. The model we present is notional and will require ongoing modification during the implementation phase.

## Limitations of the study

Given the general nature of the topic, the literature that we could have accessed was enormous and impossible to source in its entirety. The qualitative research was also potentially affected by the pre-existing assumptions and attitudes of the researchers, and the limited number of participants. This research therefore depicts a snapshot in time of a body of research that is limited by our own subjectivity, the subjectivity of the stakeholders, our interpretation of the responses and the literature we accessed. The research is thus of necessity incomplete. However, action research works well for open-ended “messy” systems such as obesity management, because the action research cycles need never end, and there is opportunity for the understandings in this work to be updated over time.

Interventions required to reverse “globesity” might need to be applied at several levels. We have focused on individual behavior change. Even if this individual MCMD approach is demonstrated to be more effective than existing approaches, it will not be sufficient to reverse global obesity [118], but will be but one part of an overall strategy.

## Abbreviations

GP: General practitioner
IOM: Institute of Medicine
MC: Multicomponent
MCMD: Multicomponent Multidisciplinary
MD: Multidisciplinary
NHMRC: National Health and Medical Research Council
QUT: Queensland University of Technology

## References

[1] Obesity and overweight [http://www.who.int/mediacentre/factsheets/fs311/en/].

[2] Excellence NIfHaC: Obesity: The prevention, identification, assessment and management of overweight and obesity in adults and children. Retrieved from https://www.nice.org.uk/guidance/cg189/evidence/obesity-update-appendix-m-pdf-6960327447; 2006. 22497033

[3] DoHa A, National Health and Medical Research Council (2013). Clinical practice guidelines for the management of overweight or obesity in adults, adolescents and children in Australia.

[4] American Dietetic Association. Adult weight management evidence-based nutrition practice guideline: American Dietetic Association Evidence Analysis Library; 2012.

[5] House of Commons HC: Obesity: Third report of session 2003–2004 (Vol. 1). Retrieved from https://publications.parliament.uk/pa/cm200304/cmselect/cmhealth/23/23.pdf; 2004.

[6] Olds T, Maher C, Zumin S, Péneau S, Lioret S, Castetbon K, Bellisle, de Wilde J, Hohepa M, Maddison R (2011). Evidence that the prevalence of childhood overweight is plateauing: data from nine countries. Int J Pediatr Obes.

[7] Ng M, Fleming T, Robinson M, Thomson B, Graetz N, Margono C, Mullany EC, Biryukov S, Abbafati C, Abera SF (2014). Global, regional, and national prevalence of overweight and obesity in children and adults during 1980-2013: a systematic analysis for the global burden of disease study 2013. Lancet.

[8] Yaskin J, Toner RW, Goldfarb N (2009). Obesity management interventions: a review of the evidence. Population Health Management.

[9] Laddu D, Dow C, Hingle M, Thomson C (2011). Going S: a review of evidence-based strategies to treat obesity in adults. Nutr Clin Pract.

[10] NHMRC: Clinical practice guidelines for the management of overweight or obesity in adults, adolescents and children in Australia. In*.* Edited by Aging DoHa. Canberra: Australian Government; 2013: 1–204.

[11] Beaulac J, Sandre D (2017). Critical review of bariatric surgery, medically supervised diets, and behavioural interventions for weight management in adults. Perspectives in Public Health.

[12] Vioque J, Ramos JM, Navarrete-Muñoz EM, García-de-la-Hera M (2010). A bibliometric study of scientific literature on obesity research in PubMed (1988-2007). Obesity Reviews: An Official Journal Of The International Association For The Study Of Obesity.

[13] Flodgren G, Deane K, Dickinson HO, Kirk S, Alberti H, Beyer FR, Brown JG, Penney TL, Summerbell CD, Eccles MP. Interventions to change the behaviour of health professionals and the organisation of care to promote weight reduction in overweight and obese adults. Cochrane Database Syst Rev. 2010;3 10.1002/14651858.CD000984.pub2 PMC4235843 20238311

[14] Roth A (2006). What works for whom?: a critical review of psychotherapy research.

[15] Dietitians Association of Australia (2012). DAA Best practice guidelines for the treatment of overweight and obesity in adults.

[16] Green L (2006). Public health asks of systems science: to advance our evidence-based practice, can you help us get more practice-based evidence?. Am J Public Health.

[17] Horn SD, DeJong G, Deutscher D (2012). Practice-based evidence research in rehabilitation: an alternative to randomized controlled trials and traditional observational studies. Archives of Physical Medicine & Rehabilitation.

[18] Parsonson BS (2012). The case for practice-based evidence to support evidence-based practice. Journal Of Primary Health Care.

[19] Avenell A, Brown TJ, McGee MA, Campbell MK, Grant AM, Broom J, Jung RT, Smith WCS (2004). What are the long-term benefits of weight reducing diets in adults? A systematic review of randomized controlled trials. Journal of Human Nutrition & Dietetics.

[20] Jakicic JM, Tate DF, Lang W, Davis KK, Polzien K, Rickman AD, Erickson K, Neiberg RH, Finkelstein EA (2012). Effect of a stepped-care intervention approach on weight loss in adults: a randomized clinical trial. JAMA.

[21] Excellence C, NICE, NICE: National Institute for Health (2014). NICE public health guidance 53. Managing overweight or obesity in adults - lifestyle weight management services.

[22] Germann JN (2009). Comprehensive multidisciplinary program perspective. Obes Manag.

[23] Kirk SFL, Penney TL, McHugh T-L, Sharma AM (2012). Effective weight management practice: a review of the lifestyle intervention evidence. Int J Obes.

[24] Grace C (2011). A review of one-to-one dietetic obesity management in adults. Journal of Human Nutrition & Dietetics.

[25] American Dietetic Association (2009). Position of the American dietetic association: weight management. J Am Diet Assoc.

[26] Hamid TKA (2009). Thinking in circles about obesity: applying systems thinking to weight management.

[27] Plsek PE, Greenhalgh T (2001). Complexity science: the challenge of complexity in health care. BMJ: British Medical Journal (International Edition).

[28] Johns DJ, Hartmann-Boyce J, Jebb SA, Aveyard P (2014). Diet or exercise interventions vs combined behavioral weight management programs: a systematic review and meta-analysis of direct comparison. J Acad Nutr Diet.

[29] Rudolph A, Hilbert A (2013). Post-operative behavioural management in bariatric surgery: a systematic review and meta-analysis of randomized controlled trials. Obes Rev.

[30] Freshwater D, Berkshire HI (2005). Action research for changing and improving practice. In: Qualitative research in health care.

[31] Finegood DT (2012). The importance of systems thinking to address obesity. Nestlé Nutrition Institute Workshop Series.

[32] Fox NJ (2003). Practice-based evidence: towards collaborative and transgressive research. Sociology.

[33] Finegood DT, Merth TDN, Rutter H (2010). Implications of the foresight obesity system map for solutions to childhood obesity. Obesity (19307381).

[34] Rao S, Perry C (2003). Convergent interviewing: a starting methodology for enterprise research programs. Qualitative Market Research.

[35] Rao S, Perry C, Hine D, Northampton CD (2007). Convergent interviewing: a starting methodology for enterprise research programs. Innovative methodologies in enterprise research. Edn.

[36] Attwater RC: Meta-methodology. In: The Sage encyclopaedia of action research edn. Edited by Coghlan D, Brydon Miller M. Los Angeles: Sage; 2014: 532–534.

[37] Dick B (2013). Convergent interviewing [On Line**]**.

[38] Driedger SM, Gallois C, Sanders CB, Santesso N (2006). Finding common ground in team-based qualitative research using the convergent interviewing method. Qual Health Res.

[39] Kelle U, Flick U, von Kardoff E, Steinke I (2004). A companion to qualitative research. Computer-assisted analysis of qualitative data.

[40] Dick B (1990). Convergent interviewing (3rd version).

[41] Jepsen DM, Rodwell JJ (2008). Convergent interviewing:a qualitative diagnostic technique for researchers. Management Research.

[42] Convergent interviewing: A technique for qualitative data collection [On line] [http://www.aral.com.au/resources/iview.html].

[43] Riege AM, Nair G (2004). The diversity of convergent interviewing applications for early researchers and postgraduate students. Mark Rev.

[44] Stringer ET, Dwyer R: Action research in human services. Upper Saddle River, New Jersey: Pearson Education; 2005.

[45] Bazeley P (2007). Qualitative data analysis with nvivo.

[46] Liamputtong P (2009). Qualitative research methods.

[47] Sankaran S, Dick B, Pasian B, Smith K (2015). Linking theory and practice in project managmenet research using action-oriented methods. Methods, Designs and Practices for Research into Project Management.

[48] Dunne C (2011). The place of the literature review in grounded theory research. Int J Soc Res Methodol.

[49] Mann T, Tomiyama AJ, Westling E, Lew A-M, Samuels B, Chatman J (2007). Medicare's search for effective obesity treatments: diets are not the answer. Am Psychol.

[50] Tsai AG, Wadden TA (2005). Systematic review: an evaluation of major commercial weight loss programs in the United States. Ann Intern Med.

[51] Tsai AG, Wadden TA (2009). Treatment of obesity in primary care practice in the United States: a systematic review. J Gen Intern Med.

[52] Waters E, de Silva-Sanigorski A, Hall BJ, Brown T, Campbell KJ, Gao Y, Armstrong R, Prosser L, Summerbell CD. Interventions for preventing obesity in children. Cochrane Database Syst Rev. 2011;12 10.1002/14651858.CD001871.pub3 22161367

[53] Loveman E, Frampton G, Shepherd J, Picot J, Cooper K, Bryant J, Welch K, Clegg A (2011). The clinical effectiveness and cost-effectiveness of long-term weight management schemes for adults: a systematic review. Health Technol Assess.

[54] Collins CE, Warren J, Neve M, McCoy P, Stokes BJ (2006). Measuring effectiveness of dietetic interventions in child obesity: a systematic review of randomized trials. Archives of Pediatrics & Adolescent Medicine.

[55] Renders CM, Valk GD, Griffin S, Wagner EH, Eijk JT, Assendelft WJ (2001). Interventions to improve the management of diabetes mellitus in primary care, outpatient and community settings. Cochrane Database Of Systematic Reviews (Online).

[56] Montes M-V, Kravitz L (2011). Unraveling the stress-eating-obesity knot: exercise can significantly mitigate the effects of stress and weight gain. IDEA Fitness Journal.

[57] Muraven M (2010). Building self-control strength: practicing self-control leads to improved self-control performance. J Exp Soc Psychol.

[58] Nauta H, Hospers HJ, Jansen A, Kok G (2000). Cognitions in obese binge eaters and obese non-binge eaters. Cogn Ther Res.

[59] Needham BL, Epel ES, Adler NE, Kiefe C (2010). Trajectories of change in obesity and symptoms of depression: the CARDIA study. Am J Public Health.

[60] Atlantis E, Ball K (2008). Association between weight perception and psychological distress. Int J Obes.

[61] Perri MG, Nezu AM, McKelvey WF, Shermer RL, Renjilian DA, Viegener BJ (2001). Relapse prevention training and problem-solving therapy in the long-term management of obesity. J Consult Clin Psychol.

[62] Sutin AR, Ferrucci L, Zonderman AB, Terracciano A. Personality and obesity across the adult life span. J Pers Soc Psychol. 2011; 10.1037/a0024286 PMC3462003 21744974

[63] Dietitians Association of Australia. Best practice guidelines for the treatment of overweight and obesity in adults. Canberra: Dietitians Association of Australia (DAA); 2005.

[64] Porter JS, Bean MK, Gerke CK, Stern M (2010). Psychosocial factors and perspectives on weight gain and barriers to weight loss among adolescents enrolled in obesity treatment. J Clin Psychol Med Settings.

[65] National Heart Lung and Blood Institute (2000). The practical guide: identification, evaluation. And treatment of overweight and obesity in adults: National Institutes of Health.

[66] Kohn M, Rees JM, Brill S, Fonseca H, Jacobson M, Katzman DK, Loghmani ES, Neumark-Sztainer D, Schneider M (2006). Preventing and treating adolescent obesity: a position paper of the Society for Adolescent Medicine. J Adolesc Health.

[67] DoHa A, National Health and Medical Research Council (2012). Australian dietary guidelines. In.

[68] Sung-Chan P, Sung YW, Zhao X, Brownson RC (2013). Family-based models for childhood-obesity intervention: a systematic review of randomized controlled trials. Obes Rev.

[69] National Health and Medical Research Council: Clinical practice guidelines for the management of overweight and obesity in adults. In*.*: Commonwealth of Australia: National Health and Medical Research Council; 2003a.

[70] National Health and Medical Research Council (2012). NHMRC. Management of overweight and obesity in adults, adolescents and children: clinical practice guidelines for primary care health professionals (draft public consultation document).

[71] Turk MW, Yang K, Hravnak M, Sereika SM, Ewing LJ, Burke LE (2009). Randomized clinical trials of weight loss maintenance: a review. J Cardiovasc Nurs.

[72] Shaw KA, Gennat HC, O'Rourke P, Del Mar C: Exercise for overweight or obesity (review). In: The Cochrane Library. Vol. issue 1, 21.01.09 edn: Wiley; 2009: 1–108. 10.1002/14651858.CD003817.pub3 PMC9017288 17054187

[73] Avenell A, Brown TJ, McGee MA, Campbell MK, Grant AM, Broom J, Jung RT, Smith WCS (2004). What interventions should we add to weight reducing diets in adults with obesity? A systematic review of randomized controlled trials of adding drug therapy, exercise, behaviour therapy or combinations of these interventions. Journal of Human Nutrition & Dietetics.

[74] Jeffery RW, Epstein LH, Wilson GT, Drewnowski A, Stunkard AJ, Wing RR (2000). Long-term maintenance of weight loss: current status. Health Psychol.

[75] World Health Organisation. Expert report on diet, nutrition and the prevention of chronic diseases: report of the joint WHO/FAO expert consultation: Geneva: WHO; 2003. p. 1–149.

[76] National Health and Medical Research Council (2010). A ‘state of the knowledge’ assessment of comprehensive interventions that address the drivers of obesity: Final report, a rapid assessment.

[77] Butland B, Jebb S, Kopelman P, Thomas S, Mardell J, Parry V (2007). Tackling obesities: future choices - foresight project report.

[78] Bulik CM (2013). Midlife eating disorders: your journey to recovery.

[79] Hay PJ, Mond J, Buttner P, Darby A (2008). Eating disorder behaviors are increasing: findings from two sequential community surveys in South Australia. PLoS Med.

[80] Maddah M, Karandish M (2011). Gender difference in obesity management among Iranian patients with metabolic syndrome. Int J Cardiol.

[81] Vernay M, Malon A, Oleko A, Salanave B, Roudier C, Szego E, Deschamps V, Hercberg S, Castetbon K (2009). Association of socioeconomic status with overall overweight andcentral obesity in men and women: the French nutrition and HealthSurvey 2006. BMC Public Health.

[82] Inelmen E, Toffanello E, Enzi G, Sergi G, Coin A, Busetto L, Manzato E (2008). Differences in dietary patterns between older and younger obese and overweight outpatients. The Journal of Nutrition Health and Aging.

[83] Duncan BL, Miller SD, Wampold BE, Hubble MA, editors. The heart and soul of change: what works in therapy. 2nd ed: Washington American psychological association; 2009.

[84] Kreindler SA, Dowd DA, Dana Star N, Gottschalk T (2012). Silos and social identity: the social identity approach as a framework for understanding and overcoming divisions in health care. Milbank Q.

[85] McNair R (2005). Breaking down the silos: Interprofessional education and inter-professionalism for an effective rural health care workforce. National Rural Health Conference (9th) Alice Springs.

[86] Bammer G (2005). Integration and implementation sciences: building a new specialization. Ecol Soc.

[87] Duncan BL, Miller SD, Sparks JA (2004). The heroic client.

[88] Taber BJ, Leibert TW, Agaskar VR (2011). Relationships among client–therapist personality congruence, working alliance, and therapeutic outcome. Psychotherapy.

[89] Sharf J, Primavera LH, Diener MJ (2010). Dropout and therapeutic alliance: a meta-analysis of adult individual psychotherapy. Psychother Theory Res Pract Train.

[90] Abramson R, Garg M, Meghreblian MJ (1980). Behavior modification for obesity: effect of therapist-patient relationship. Psychosomatics.

[91] Wagner EH, Austin BT, Davis C, Hindmarsh M, Schaefer J, Bonomi A (2001). Improving chronic illness care: translating evidence into action: interventions that encourage people to acquire self-management skills are essential in chronic illness care. Health Aff.

[92] Institute of Medicine (2001). Crossing the quality chasm: a new health system for the 21st century.

[93] McGowan PT (2012). Self-management education and support in chronic disease management. Primary Care.

[94] Department of Health (2011). WA chronic conditions self-management strategic framework 2011–2015.

[95] Lambert MJ, Garfield SL, Bergin AE: Overview, trends and future issues. In: Bergin and Garfield’s handbook of psychotherapy and behavior change 5th edn. Edited by Lambert MJ. New York Wiley; 2004: 805–819.

[96] Oandasan I, Baker GR, Barker K, Bosco C, D’Amour D, Jones L, Kimpton S, Lemieux-Charles L, Nasmith L, San Martin Rodriguez L (2006). Teamwork in healthcare: promoting effective teamwork in healthcare in Canada.

[97] Dick B (1991). Helping groups to be effective: skills, processes and concepts for group facilitation.

[98] Bovet P, Gervasoni J-P, Mkamba M, Balampama M, Lengeler C, Paccaud F (2008). Go4it; study design of a randomised controlled trial and economic evaluation of a multidisciplinary group intervention for obese adolescents for prevention of diabetes mellitus type 2. BMC Public Health.

[99] Donini LM, Savina C, Castellaneta E, Coletti C, Paolini M, Scavone L, Civale C, Ceccarelli P, Zaninotto S, Tineri M (2009). Multidisciplinary approach to obesity. Eating And Weight Disorders: EWD.

[100] Orchard CA, Curran V, Kabene S (2005). Creating a culture for interdisciplinary collaborative professional practice. Medical Education Online.

[101] Ham C, Dixon A, Brooke B (2012). Transforming the delivery of health and social care: the case for fundamental change.

[102] Swinburn B, Sacks G, Hall K, McPherson K, Finegood D, Moodie M, Gortmaker S (2011). The global obesity pandemic: shaped by global drivers and local environments. Lancet.

[103] Power ML (2012). The human obesity epidemic, the mismatch paradigm, and our modern “captive” environment. American Journal of Human Biology: The Official Journal of the Human Biology Council.

[104] Vandenbroeck P, Goossens J, Clemens M (2007). Foresight: tackling obesities: future choices - building the obesity system map.

[105] Ulijaszek SJ (2015). With the benefit of foresight: obesity, complexity and joine-up government. BioSocieties.

[106] Rutter H (2012). The single most important intervention to tackle obesity. International Journal of Public Health.

[107] Kelly SA, Melnyk BM (2008). Systematic review of multicomponent interventions with overweight middle adolescents: implications for clinical practice and research. Worldviews Evid-Based Nurs.

[108] Tsui A. Spirit of science and socially responsible scholarship: Manag Organ Rev; 2013.

[109] Glaser BG (1992). Basics of grounded theory analysis: emergence vs. forcing.

[110] Dick B (2003). What can action researchers learn from grounded theorists?.

[111] McIntyre A (2008). Participatory action research.

[112] Brownlie D, Hewer P, Wagner B, Svensson G (2008). Management theory and practice: bridging the gap through multidisciplinary lenses. Eur Bus Rev.

[113] Cooper Z, Fairburn CG, Hawker DM (2004). Treatment of obesity: a clinician's guide.

[114] Stubbs J, Whybrow S, Teixeira P, Blundell J, Lawton C, Westenhoefer J, Engel D, Shepherd R, McConnon Á, Gilbert P (2011). Problems in identifying predictors and correlates of weight loss and maintenance: implications for weight control therapies based on behaviour change. Obes Rev.

[115] Egger G, Binns A, Rossner S (2008). Lifestyle medicine.

[116] Ford ES, Mokdad AH (2008). Epidemiology of obesity in the western hemisphere. J Clin Endocrinol Metab.

[117] Enwald HPK, Huotari M-LA: Preventing the obesity epidemic by second generation tailored health communication: an interdisciplinary review. J Med Internet Res 2010, 12(2):16–16. 10.2196/jmir.1409 PMC2956235 20584698

[118] Gortmaker SL, Swinburn BA, Levy D, Carter R, Mabry PL, Finegood DT, Huang T, Marsh T, Moodie ML (2011). Changing the future of obesity: science, policy, and action. Lancet.

[119] National Health and Medical Research Council (2007). National statement on ethical conduct in human research.

